# Reactive Adsorption Performance and Behavior of Gaseous Cumene on MCM-41 Supported Sulfuric Acid

**DOI:** 10.3390/molecules27165129

**Published:** 2022-08-11

**Authors:** Dandan Zhao, Yuheng Liu, Xiaolong Ma, Jinjin Qian, Zichuan Ma

**Affiliations:** 1 Hebei Key Laboratory of Inorganic Nano-Materials, College of Chemistry and Material Sciences, Hebei Normal University, Shijiazhuang 050024, China; 2 Hebei Key Laboratory of Innovative Drug Research and Evaluation, College of Pharmaceutical Sciences, Hebei Medical University, Shijiazhuang 050017, China; 3 School of Environmental Science and Engineering, Hebei University of Science and Technology, Shijiazhuang 050018, China

**Keywords:** supported sulfuric acid, MCM-41, cumene, reactive adsorption, sulfonation

## Abstract

Efficient removal of cumene from gaseous streams and recovery of its derivatives was accomplished using a MCM-41-supported sulfuric acid (SSA/MCM-41) adsorbent. The results indicated that the removal performance of the SSA/MCM-41 for cumene was significantly influenced by the process conditions such as bed temperature, inlet concentration, bed height, and flow rate. The dose–response model could perfectly describe the collected breakthrough adsorption data. The SSA/MCM-41 adsorbent exhibited a reactive temperature region of 120–170 °C, in which the cumene removal ratios (*X*_c_) were greater than 97%. Rising the bed height or reducing the flow rate enhanced the theoretical adsorption performance metrics, such as theoretical breakthrough time (*t*_B,th_) and theoretical breakthrough adsorption capacity (*Q*_B,th_), whereas increasing the inlet concentration resulted in *t*_B,th_ shortening and *Q*_B,th_ rising. As demonstrated in this paper, the highest *t*_B,th_ and *Q*_B,th_ were 69.60 min and 324.50 mg g^−1^, respectively. Meanwhile, the spent SSA/MCM-41 could be desorbed and regenerated for cyclic reuse. Moreover, two recoverable adsorbed products, 4-isopropylbenzenesulfonic acid and 4, 4′-sulfonyl bis(isopropyl-benzene), were successfully separated and identified using FTIR and ^1^H/^13^C NMR characterization. Accordingly, the relevance of a reactive adsorption mechanism was confirmed. This study suggests that the SSA/MCM-41 has remarkable potential for application as an adsorbent for the resource treatment of cumene pollutants.

## 1. Introduction

Volatile organic compounds (VOCs) have a significant role in the formation of secondary organic aerosols and the destruction of atmospheric ozone. Moreover, their toxicity and pathogenicity are also constantly warned [1,2,3]. Among all, aromatics are well recognized as highly polluting and toxic VOCs, the emission of which has been subjected to strict legal restrictions [4,5,6,7]. Therefore, the purification of aromatics has attracted more and more attention. 

Among various traditional methods for VOCs purification, destructive purification techniques, such as catalytic oxidation [4,8,9,10], thermal combustion [11], photocatalytic degradation [2,12,13], and biodegradation [14], have achieved excellent mineralization rates for VOCs without secondary pollution, but their efficient utilization is often forgone. While physical treating techniques, such as condensation [15], absorption [16], adsorption [17,18,19], and membrane separation [20], attain recycling of the original VOC molecules to a limited degree under certain conditions, they readily lead to secondary pollution or exhibit low removal efficiencies. To achieve efficient treatment of VOCs, our group developed a novel reactive adsorption approach that employs supported sulfuric acid for specifically removing the aromatic hydrocarbons [21]. The feasibility and efficiency of the approach were verified in the treatment of an *o*-xylene simulated waste gas stream [21,22,23,24]. By inferring from the interfacial sulfonation mechanism revealed in the abovementioned studies, the reactive adsorption performance is most probably linked to the structures of the benzene-containing compounds. Therefore, cumene (isopropyl benzene) with a large, substituted alkyl group that poses the electron-donating and steric hindrance effects was selected as a model phenyl pollutant in this study to facilitate the application and further improvement of this novel approach. The underlying objectives of this work were (1) to evaluate the reactive activity of cumene on MCM-41 supported sulfuric acid (SSA/MCM-41) with increasing temperature; (2) to experimentally examine and theoretically fit the breakthrough data for cumene removal in an SSA/MCM-41 fixed-bed column with adjusted inlet concentration (*C*_in_), bed height (*h*), and flow rate (ν_g_) to investigate the cumene adsorption behavior; and (3) to characterize the adsorbed species to explore the mechanism underlying cumene removal from the gaseous stream. We believe that the obtained results will be helpful to further understand the treatment potential of this novel gas–solid adsorption approach for benzene derivatives.

## 2. Results

### 2.1. Effects of Temperature on Cumene Reaction-Type Adsorption

**Figure 1:**
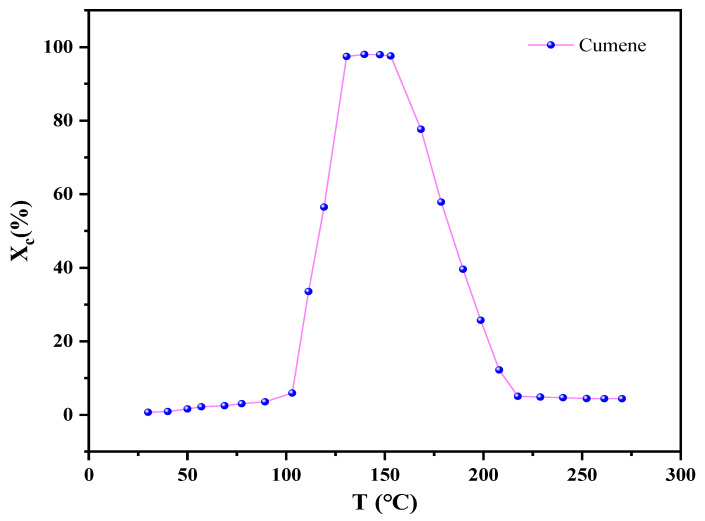
Plot of cumene removal ratios (*X*_c_) vs. temperature (*T*).

As shown in Figure 1, the cumene removal ratios vs. temperature (*X*_c_ − *T*) curve presented an inverted U-shaped profile, indicating that the temperature range of 120–170 °C was apt for the reactive adsorption of cumene on MCM-41-supported sulfuric acid (SSA/MCM-41). The two sides of the U-shaped profile represent the activation (upward trend) and the deactivation regions (downward trend). This adsorption behavior is similar to *o*-xylene on various supported sulfuric acid systems such as SSA/MCM-41, SSA/SBA-15, SSA/SG [22], and two synthesized silica-supported sulfuric acid (SSA I and SSA II) samples [23]. As a result, resembling *o*-xylene, cumene was likely removed from the gas stream by the sulfonation reaction with the anchored H_2_SO_4_ molecules on SSA/MCM-41. Therefore, the cumene removal process was similarly referred to as reactive adsorption. 

**Figure 2:**
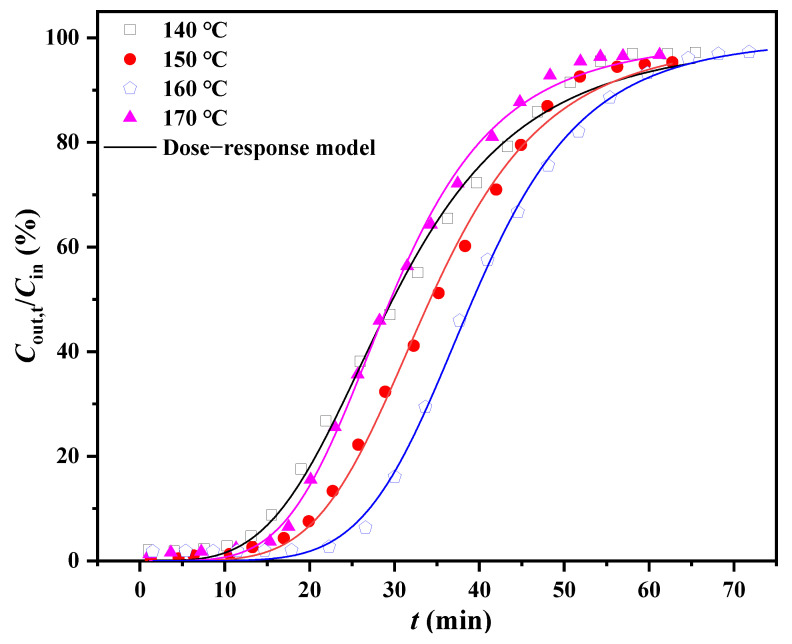
Experimental dotted lines and dose–response model fitting breakthrough curves at different bed temperatures.

In order to further understand the temperature dependence of SSA/MCM-41 in the reactive adsorption of cumene, dotted plots were developed based on the dose–response model-fitting curves. These curves help describe the dynamic adsorption breakthrough behavior at different bed temperatures, as depicted in Figure 2. The breakthrough profiles presented an S-shaped behavior, indicating that the interaction between the cumene gas stream and the SSA/MCM-41-filled bed can be divided into three stages: an early phase of highly efficient reactive adsorption (*X*_c_ ≥ 95%), a middle breakthrough phase of rapidly decaying adsorption (5% < *X*_c_ < 95%), and an ending phase of inefficient reactive adsorption (*X*_c_ ≤ 5%). This behavior is consistent with typical physisorption or chemisorption in gas–solid systems [25,26,27]. It can also be seen from Figure 2 that with the temperature increasing, the middle breakthrough phase of the curves shifted first to the right-hand side and then to the left-hand side, thus rendering an optimum temperature of 160 °C, which was probably due to the opposite effects of temperature increase on the sulfonation reaction kinetics and the stability of supported sulfuric acids [22]. Furthermore, the observed magnitudes of the adsorption performance metrics are presented in Table 1, which reflect how the temperature affected the reactive adsorption performance of SSA/MCM-41 for cumene quantitatively. The corresponding values at 160 °C were 24.68 min and 222.41 mg g^−1^ for the *t*_B_ and *Q*_B_ metrics, respectively.

An excellent correlation was demonstrated for the dose–response model fitted to the experimental breakthrough data at different bed temperatures (Figure 2), with a fitting correlation coefficient (*R*^2^) higher than 0.995 (Line 7 in Table 1). Furthermore, both theoretical metrics *t*_B,th_ and *Q*_B,th_ estimated by this model were very close to the experimental values, indicating that the model has been very suitable for describing the dynamics of the reactive adsorption process of cumene. According to the prediction of the dose–response model, the experiment at 160 °C demonstrated a top *Q*_B,th_ of 223.13 mg·g^−1^.

### 2.2. Comprehensive Analysis of Effects of Inlet Concentration, Bed Height, and Flow Rate

**Figure 3:**
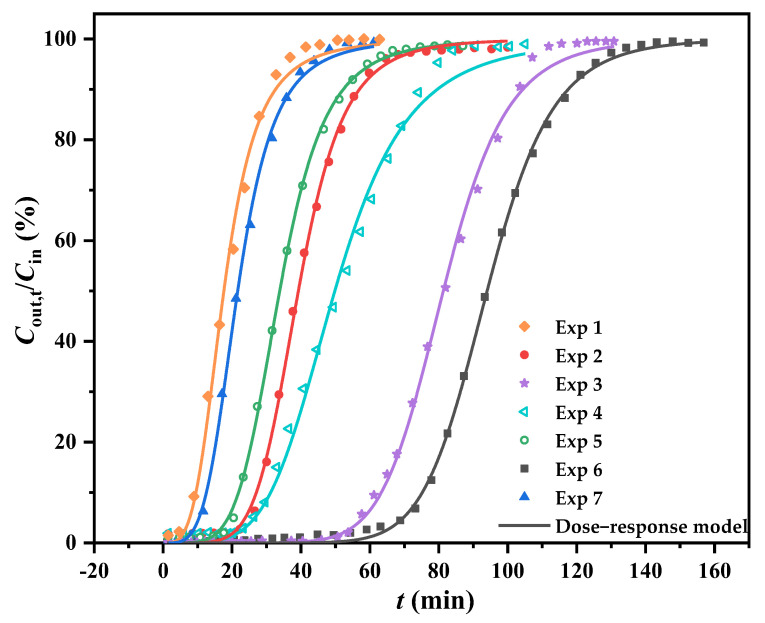
Experimental dotted plots and dose–response model-fitting breakthrough curves for the seven experiments.

Under the optimum temperature of 160 °C, the cumene breakthrough curves in the SSA/MCM-41-filled bed were studied at different process conditions (Table 2) to analyze the effects of inlet concentration (*C*_in_), gas flow rate (*v*), and bed height (*h*) on the cumene adsorption performance. The experimental dotted plots and the corresponding dose–response model-fitting curves obtained from the seven experiments are shown in Figure 3. The experimental and model-fitting results are reported in Table 3. The main findings are discussed in what follows.

(1) All breakthrough profiles presented typical S-like curves, indicating the presence of a strong interaction between the cumene molecules and the SSA/MCM-41 surface, attributable to their sulfonation reaction (see the next section).
(2) At the stable conditions of *v* = 50 mL min^−1^ and *C*_in_ = 9.2 mg L^−1^, when *h* rose from 10.5 to 20.0 mm (Exp. nos. 1–3), the curves shifted significantly to the right-hand side, the *t*_B,th_ was highly extended, and the *Q*_B,th_ values increased, indicating that a prolonged residence time could improve the total removal amount of cumene and enhance its specific capacity. This finding was backed up by another set of experiments (Exp. nos. 7, 2, and 6) in which the residence time was prolonged by reducing *v* from 75 to 25 mL min^−1^ under the same conditions of *C*_in_ = 9.2 mg L^−1^ and *h* = 14.3 mm. These experiments demonstrated the same response trend regarding both *t*_B,th_ and *Q*_B,th_ metrics.
(3) When controlling the residence time at the same value in the third experiment, changing *C*_in_ from 5.3 to 14.6 mg L^−1^ (Exp. nos. 4, 2, and 5) resulted in *t*_B,th_ shortening and increased *Q*_B,th_. These changes could be attributed to the relative shortage of the SSA adsorption sites and the enhancement of cumene reaction driving force caused by the *C*_in_ increase [28].
(4) In the experiments mentioned above, it was found that both maximum values of *t*_B,th_ (69.60 min) and *Q*_B,th_ (324.50 mg g^−1^) were obtained in Exp. No. 6, and the corresponding optimal process conditions were 9.2 mg L^−1^ for *C*_in_, 14.3 mm for *h*, 25 mL min^−1^ for *v*, and 160 °C for *T*. The Q_B,th_ value identified that SSA/MCM-41 outperformed a silica-alumina material (0.82 mg g^−1^; 60 °C) and activated carbon (230.25 mg g^−1^; 20.8 °C) on the adsorption capacity of cumene [29,30], which could be related to the sulfonic acid loading on MCM-41.

### 2.3. Identification of the Adsorbed Products and Removal Mechanism for Cumene

**Scheme 1:**
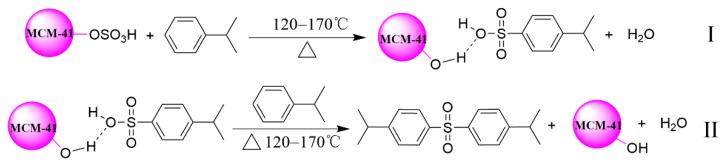
Production mechanism of 4-isopropylbenzenesulfonic acid (**I**) and 4,4’-sulfonyl-bis(isopropylbenzene) (**II**) in the cumene removal process.

As further described in Section 3.5, two amorphous white solids could be separated out from the spent SSA/MCM-41, implying that two adsorbed products (I and II) were formed during the reactive adsorption of cumene on SSA/MCM-41. The results of FTIR and ^1^H/^13^C NMR for the adsorbed products I and II and their mixture products are depicted in Figures S1–S9. The spectral data for the adsorbed products are summarized in Table 4. The results confirmed that the adsorbed product I was 4-isopropylbenzenesulfonic acid and adsorbed product II was 4, 4′-sulfonyl bis(isopropyl-benzene). In addition, according to the results of ^1^H NMR characterization of the mixture products obtained (Figure S8) and the calculated data from the Formula (S-1), it can be confirmed that the content ratio of product I to product II was 12:1. Therefore, it can be concluded that the cumene removal from the gaseous stream by SSA/MCM-41 was by reactive adsorption with the mechanism depicted in Scheme 1. This adsorption process involves the conversion of cumene to sulfonic acid and sulfone derivatives with higher molecular weights and polarities that can be strongly adsorbed onto the MCM-41 surface.

**Figure 4:**
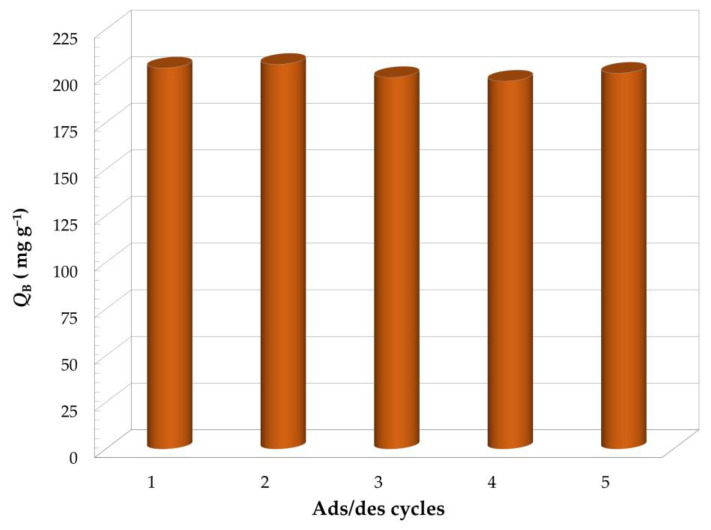
Breakthrough adsorption capacity of regenerated SSA/MCM-41 for cumene.

The experiments demonstrated that ethanol extraction could separate the adsorbed products (I and II) and the MCM-41 support from the spent SSA/MCM-41. Thus, it enabled recovering the adsorbed products and regenerating the support. The regenerated MCM-41 could be reused to prepare the novel SSA/MCM-41 via wet impregnation. Figure 4 presents the adsorption/regeneration cyclic analysis for cumene removal by SSA/MCM-41. The *Q*_B_ values of the four successive regeneration cycles of SSA/MCM-41 did not show a significant loss in comparison with the fresh counterpart, demonstrating that the SSA/MCM-41 possessed robust stability and reusability in the raw stream treatment application of cumene.

## 3. Experimental Section 

### 3.1. Materials

The chemicals and reagents used in the study were of analytical grade, which included sulfuric acid (98%, Tianjin Pharmaceutical Chemical Co., Ltd., Tianjin, China) and cumene (99%, Shanghai Yien Chemical Technology Co., Ltd., Shanghai, China). In addition, MCM-41 (28–60 nm) was purchased from Nanjing Nanotechnology Co., Ltd., Nanjing, China.

### 3.2. Preparation of Supported Sulfuric Acid

The supported sulfuric acid, SSA/MCM-41, was typically prepared by wet impregnation in an appropriate volume of sulfuric acid solution. Specifically, 8 g of MCM-41 was pulverized and passed through a 0.15 mm sieve and was impregnated with 14.8 mL of 5.0 mol L^−1^ sulfuric acid solution at room temperature for 1 h. The sample was then dried in an oven at 80 °C for 48 h to obtain an adsorbent with sulfuric acid loading of 9.25 mmol g^−1^, which was regarded as an optimized sample identified from the preliminary studies [24]. Finally, the prepared SSA/MCM-41 was stored in a desiccator for further use.

### 3.3. Characterization

The adsorbed products were characterized by two techniques. First, Fourier-transform infrared spectra (FTIR, Bruker AXS TENSOR 27, Brucker, Karlsruhe, Germany) based on the KBr tablet method were employed to identify the molecular composition. Proton and carbon nuclear magnetic resonances (^1^H NMR and ^13^C NMR; Bruker AVIII500, Brucker, Karlsruhe, Germany) were performed to confirm the molecular structure of the adsorbed products.

### 3.4. Reactive Adsorption Tests

Reactive adsorption tests for removing gaseous cumene were carried out in a continuous flow glass column (6 mm i.d. × 40 cm length). SSA/MCM-41 (50 mg, ≤0.15 mm) was mixed with quartz sand (100 mg, 0.180–0.425 mm) and packed into the glass column reactor with inert glass fiber plugs at both ends. The gas flow rate was controlled with a mass flowmeter (SevenStar, D07-26), and the cumene concentration was monitored on a GC7900 gas chromatograph (Tianmei, Hangzhou, China) equipped with a flame ionization detector (FID) and a polyethylene glycol (PEG)-20M capillary column (30 m × 0.32 mm). In order to explore the adsorption behavior and performance of SSA/MCM-41 for cumene pollutants, a three-pronged study was performed:

(1) The removal rate of cumene (*X*_c_) was measured while heating gradually from room temperature up to 300 °C at 5 °C min^−1^ under *v* = 50 mL min^−1^, *C*_in_ = 9.2 mg L^−1^, and *h* = 14.3 mm. First, the *X*_c_ values were calculated via Equation (1). Then, the values were used to plot an *X*_c_ − *T* curve to analyze the reactive adsorption behavior of cumene upon the variation of the adsorption bed temperature.
(2) A series of transient adsorption experiments for the cumene/SSA/MCM-41 system was performed by independently changing the bed height (*h*), flow rate (*v*), or inlet concentration (*C*_in_) with the other two variables kept constant (see Table 2). The outlet concentrations of cumene (*C*_out,t_, mg L^−1^) in the gas flow at time *t* were determined experimentally. The extensive data collected were treated to draw the experimental breakthrough adsorption curves. These curves were then used to demonstrate the reactive behavior of SSA/MCM-41 toward cumene adsorption at various process conditions and to determine the experimental adsorption performance metrics such as breakthrough time (*t*_B_, min) and breakthrough adsorption capacity (*Q*_B_, mg·g^−1^). Here, *t*_B_ was the time corresponding to the ratio *C*_out,t_/*C*_in_ = 0.05 on the curve, and *Q*_B_ was calculated from Equation (2).

(1) $$X_{C}=\frac{C_{\mathrm{in}}-C_{\mathrm{out},t}}{C_{\mathrm{in}}}\times 100\%$$

(2) $$Q_{B}=\frac{V_{g}C_{\mathrm{in}}}{m}\int_{0}^{t_{B}}\left(1-\frac{C_{\mathrm{out},t}}{C_{\mathrm{in}}}\right)dt,$$

(3) Based on our previous work [23,29], the dose–response model (Equation (3)) was selected to fit the experimental data to more deeply understand the adsorption behavior of cumene in the SSA/MCM-41 fixed-bed column and predict the theoretical adsorption performance metrics (*t*_B,th_/min and *Q*_B,th_/mg·g^−1^).

(3) $$\frac{C_{\mathrm{out},t}}{C_{\mathrm{in}}}=1-\frac{1}{1+(\frac{C_{\mathrm{in}}\times \nu \times t}{{q0}_{\;}\times \;m}{)}^{a}},$$

where *q*_0_ and *a* are constants of the dose–response model.

### 3.5. Desorption and Regeneration of the Spent SSA/MCM-41

The SSA/MCM-41 sample exposed to cumene reactive adsorption is labeled as the spent adsorbent. First, the spent SSA/MCM-41 (5 g) was extracted with 20 mL absolute ethanol at room temperature. Then the product formed in the adsorption process was extracted by rotating evaporation of the extracted solution. In the second step, the solid filtered out from the previous step was washed with distilled water and dried at 100 °C to attain the regenerated MCM-41, which was reused for preparing the SSA/MCM-41 according to the same procedure described above. Next, the product obtained in the first step was dissolved in a small amount of water and then extracted with ethyl acetate (20 mL × 3). The resulting organic phase was dried with anhydrous sodium sulfate overnight, after which the solvent was filtered and concentrated. After that, the product was purified by silica gel column chromatography using a mixture of petroleum ether and ethyl acetate (V_1_:V_2_ = 3:1) as eluent. Finally, the structures of the products were analyzed by FTIR and ^1^H/^13^C NMR.

## 4. Conclusions

The SSA/MCM-41 sample with sulfuric acid loading of 9.25 mmol g^−1^ was prepared by the wet impregnation method and used as an adsorbent for the treatment of cumene waste gas. The results of the temperature response tests demonstrated that the SSA/MCM-41 adsorbent exhibited a reactive temperature range of 120–170 °C, in which *X*_c_ were greater than 97%, where the optimum temperature was 160 °C. It was found that the dose–response model could perfectly describe the breakthrough adsorption data. Comprehensive analysis of the effects of process conditions marked the importance of cumene inlet concentration, bed height, and flow rate. Rising the bed height and reducing the flow rate enhanced cumene removal performance in *t*_B,th_ and *Q*_B,th_, whereas increasing the inlet concentration resulted in *t*_B,th_ shortening and *Q*_B,th_ rising. As a result, the highest *t*_B,th_ and *Q*_B,th_ for cumene adsorption on SSA/MCM-41 were determined to be 69.60 min and 324.50 mg g^−1^, respectively. Furthermore, FTIR and ^1^H/^13^C NMR results confirmed that the adsorbed products included both 4-isopropylbenzenesulfonic acid and 4, 4′-sulfonyl bis(isopropyl-benzene). Therefore, it can be concluded that cumene removal from the gaseous stream by SSA/MCM-41 followed a reactive adsorption mechanism, which combined the adsorption with sulfonation reactions between the cumene and anchored H_2_SO_4_. In addition, the spent SSA/MCM-41 could be desorbed and regenerated for repeated use.

## Figures and Tables

**Table 1:** Experimental and model-fitting results of different bed temperatures.

Parameters	Temperature (°C)			
	140	150	160	170
Experimental test:				
*t*_B_ (min)	13.11	16.23	24.68	14.12
*Q*_B_ (mg g^−1^)	117.30	146.81	222.41	127.13
Dose–response model:				
*q*_0_ (mg g^−1^)	0.27	0.31	0.36	0.27
*a*	3.81	4.89	5.90	4.57
*R* ^2^	0.995	0.997	0.999	0.998
*t*_B,th_ (min)	13.71	18.71	24.87	15.50
*Q*_B__,th_ (mg g^−1^)	172.42	218.79	223.13	198.62

**Table 2:** Summary of the experimental conditions.

Exp. No.	Inlet Concentration (mg L^−1^)	Bed Height (mm)	Flow Rate (mL min^−1^)
1 (◆)	9.2	10.5	50
2 (●)	9.2	14.3	50
3 (★)	9.2	20.0	50
4 (◁)	5.3	14.3	50
5 (○)	14.6	14.3	50
6 (■)	9.2	14.3	25
7 (▲)	9.2	14.3	75

**Table 3:** Adsorption performance metrics of cumene at different conditions.

Metrics	Exp. No.						
	1	2	3	4	5	6	7
*t*_B,th_ (min)	6.73	24.87	58.06	26.56	16.25	69.60	10.42
*Q*_B__,th_ (mg g^−1^)	118.31	223.13	268.55	140.80	231.43	324.50	151.59

**Table 4:** ^1^H/^13^C NMR and FTIR results of adsorbed products I and II.

Products	Methods & Parameters	The Data for Adsorbed Products
I	^1^H NMR (DMF, 500 MHz), δ ppm	7.51 (d, *J* = 5.0 Hz, 2 H), 7.18 (d, *J* = 5.0 Hz, 2 H), 3.33 (s), 2.88 (m, 1 H), 1.19 (d, *J* = 2.5 Hz, 6 H)
	^13^C NMR (DMF, 125 MHz), δ ppm	23.82 (2 C), 33.18, 125.39 (2 C), 125.53 (2 C), 137.35, 148.55
	FTIR (KBr), cm^−1^	3426, 2961, 2869, 1629, 1465, 1408, 1180, 1052, 1004, 831, 769, 677, 571
II	^1^H NMR (DMF, 500 MHz), δ ppm	7.85 (d, *J* = 5.0 Hz, 2 H), 7.33 (d, *J* = 2.5 Hz, 2 H), 2.94 (m, 1 H), 1.23 (d, *J* = 2.5 Hz, 6 H)
	^13^C NMR (DMF, 125 MHz), δ ppm	23.63 (4 C), 34.22 (2 C), 127.36 (4 C), 127.78 (4 C), 139.32 (2 C), 154.59 (2 C)
	FTIR (KBr), cm^−1^	3440, 2966, 2931, 2869, 1600, 1408, 1370, 1321, 1158, 1109, 1052, 836, 788, 672, 571

## Data Availability

Data were contained within the article or Supplementary Material. The data presented in this study are available in Supplementary Materials.

## Supplementary Materials

The following are available online: https://www.mdpi.com/article/10.3390/molecules27165129/s1, Figure S1: FTIR spectrum of the adsorbed product I, Figure S2: FTIR spectrum of the adsorbed product II, Figure S3: ^1^H NMR spectrum of the adsorbed product I, Figure S4: ^13^C NMR spectrum of the adsorbed product I, Figure S5: ^1^H NMR spectrum of the adsorbed product II, Figure S6: ^13^C NMR spectrum of the adsorbed product II, Figure S7: FTIR spectrum of the adsorbed product from the resulting mixture, Figure S8: ^1^H NMR spectrum of the adsorbed product from the resulting mixtur, Figure S9: ^13^C NMR spectrum of the adsorbed product from the resulting mixture, Table S1: Experimental and fitting results on adsorption performance. Note: There were two adsorption products in the experiments. One was 4-isopropylbenzene- sulfonic acid (I) and another was 4, 4′-sulfonyl bis(isopropylbenzene) (II).

## Sample Availability

Samples of the compounds are not available from the authors.
