# Supplementary material for: Reactive Adsorption Performance and Behavior of Gaseous Cumene on MCM-41 Supported Sulfuric Acid
Source: Molecules. 2022 Aug 11;27(16):5129. doi: 10.3390/molecules27165129 (PMC9416091; doi:10.3390/molecules27165129)
Supplement: Supplementary file 1 [file molecules-27-05129-s001.zip › molecules-1831398-supplementary.pdf]

Supplementary Material

Reactive Adsorption Performance and Behavior of Gaseous Cumene on MCM-41 Supported Sulfuric Acid

Dandan Zhao [1], Yuheng Liu [2,*], Xiaolong Ma [3], Jinjin Qian [1], Zichuan Ma [2,*]

[1] Hebei Key Laboratory of Inorganic Nano-Materials, College of Chemistry and Material Sciences, Hebei Normal University, Shijiazhuang, 050024, China
[2] Hebei Key Laboratory of Innovative Drug Research and Evaluation, College of Pharmaceutical Sciences, Hebei Medical University, Shijiazhuang 050017, China
[3] School of Environmental Science and Engineering, Hebei University of Science and Technology, Shijiazhuang 050018, China
* Correspondence: 18100757@hebmu.edu.cn (Y.L.); mazc@hebtu.edu.cn (Z.M.);
Tel.: +86 0311 80787400 (Z.M.)

Contents

Figure S1. FTIR spectrum of the adsorbed product I 2
Figure S2. FTIR spectrum of the adsorbed product II 2
Figure S3. ^{1}H NMR spectrum of the adsorbed product I 3
Figure S4. ^{13}C NMR spectrum of the adsorbed product I 3
Figure S5. ^{1}H NMR spectrum of the adsorbed product II 4
Figure S6. ^{13}C NMR spectrum of the adsorbed product II 4
Figure S7. FTIR spectrum of the adsorbed product from the resulting mixture 5
Figure S8. ^{1}H NMR spectrum of the adsorbed product from the resulting mixture 5
Figure S9. ^{13}C NMR spectrum of the adsorbed product from the resulting mixture 6
Table S1. Experimental and fitting results on adsorption performance 6

Note: there were two adsorption products in the experiments. One was 4-isopropylbenzene-sulfonic acid (I), another was 4,4'- sulfonylbis(isopropylbenzene) (II).

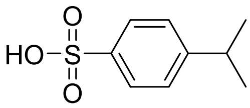

(I)

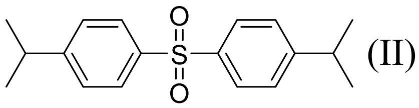

(II)

4-isopropylbenzenesulfonic acid 4,4'-sulfonylbis(isopropylbenzene)

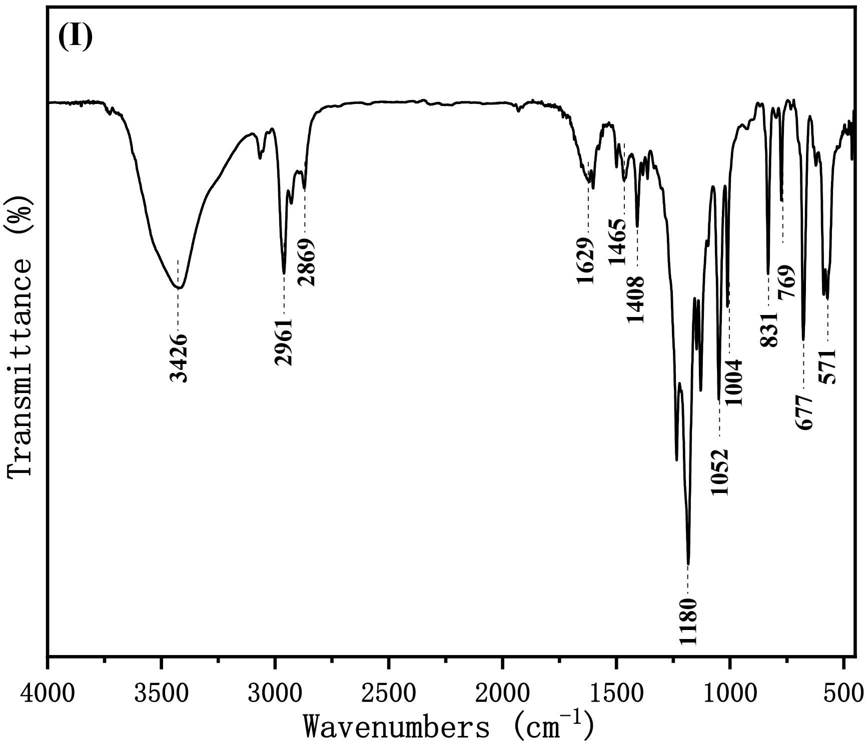

Figure S1. FTIR spectrum of the adsorbed product I

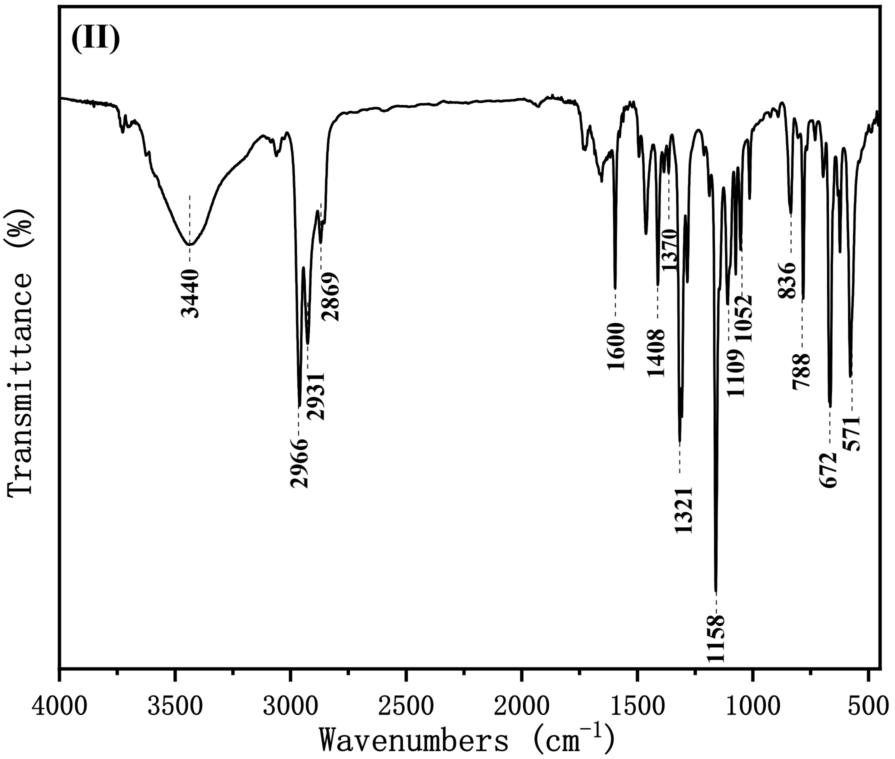

Figure S2. FTIR spectrum of the adsorbed product II

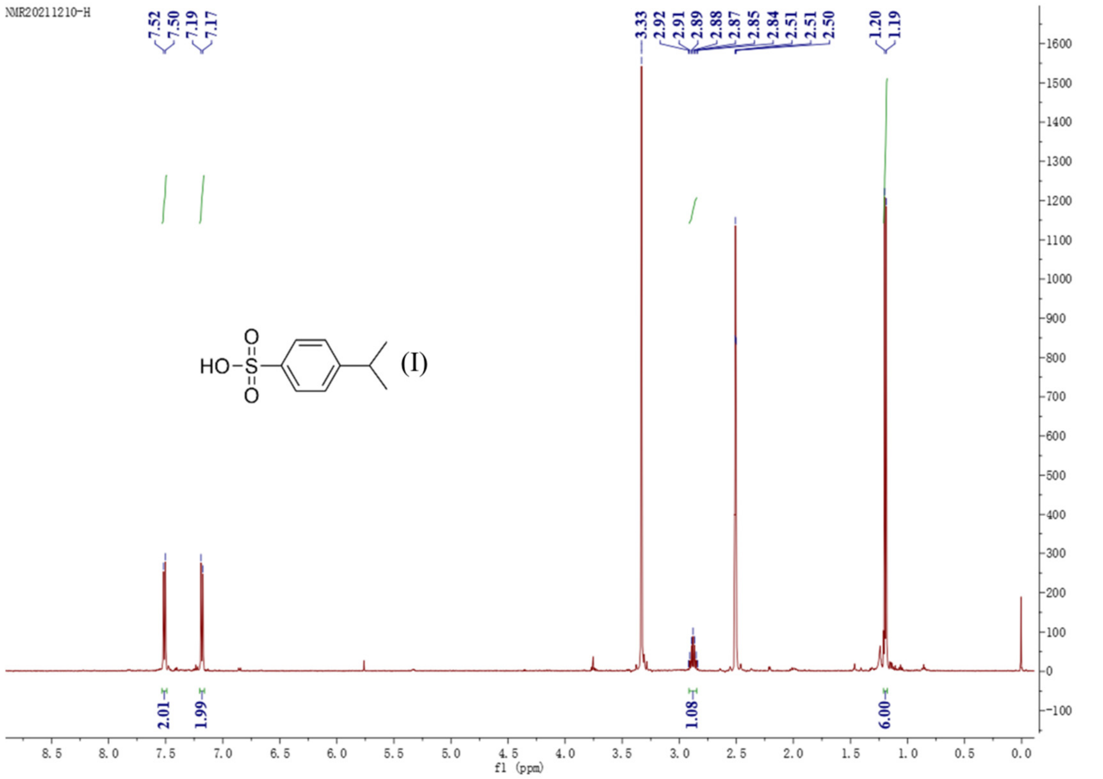

Figure S3. The ^{1}H NMR spectrum of the adsorbed product I

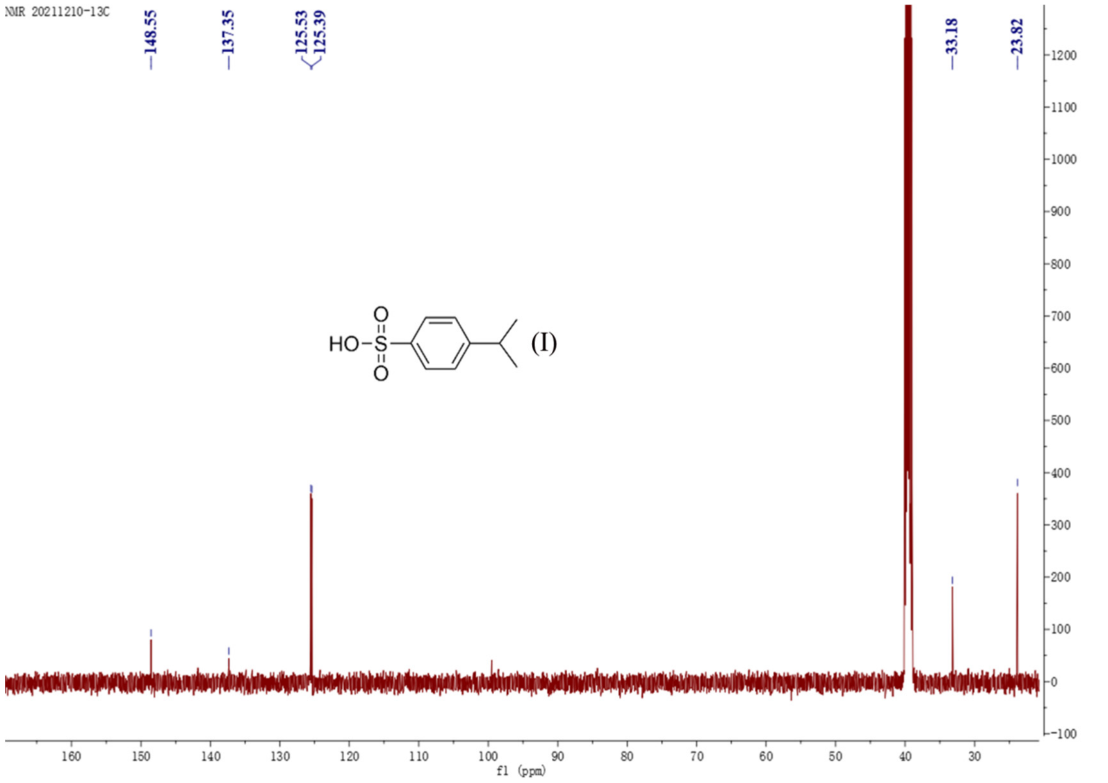

Figure S4. ^{13}C NMR spectrum of the adsorbed product I

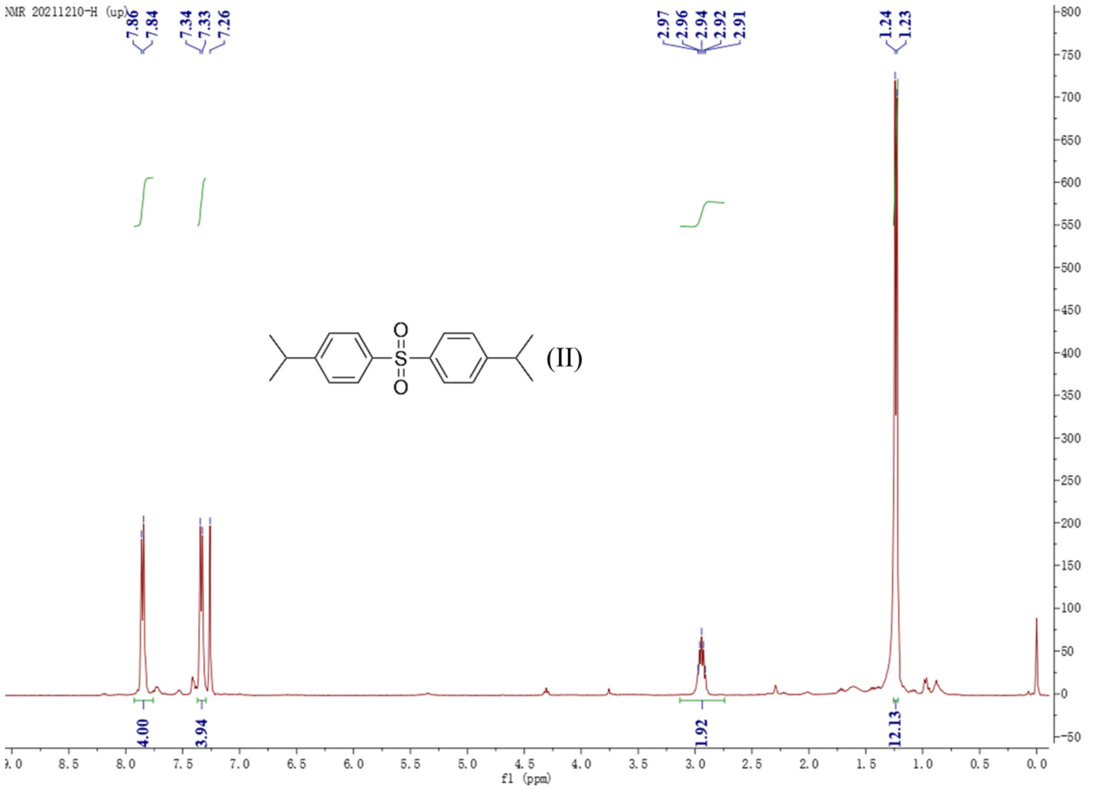

Figure S5. ^{1}H NMR spectrum of the adsorbed product II

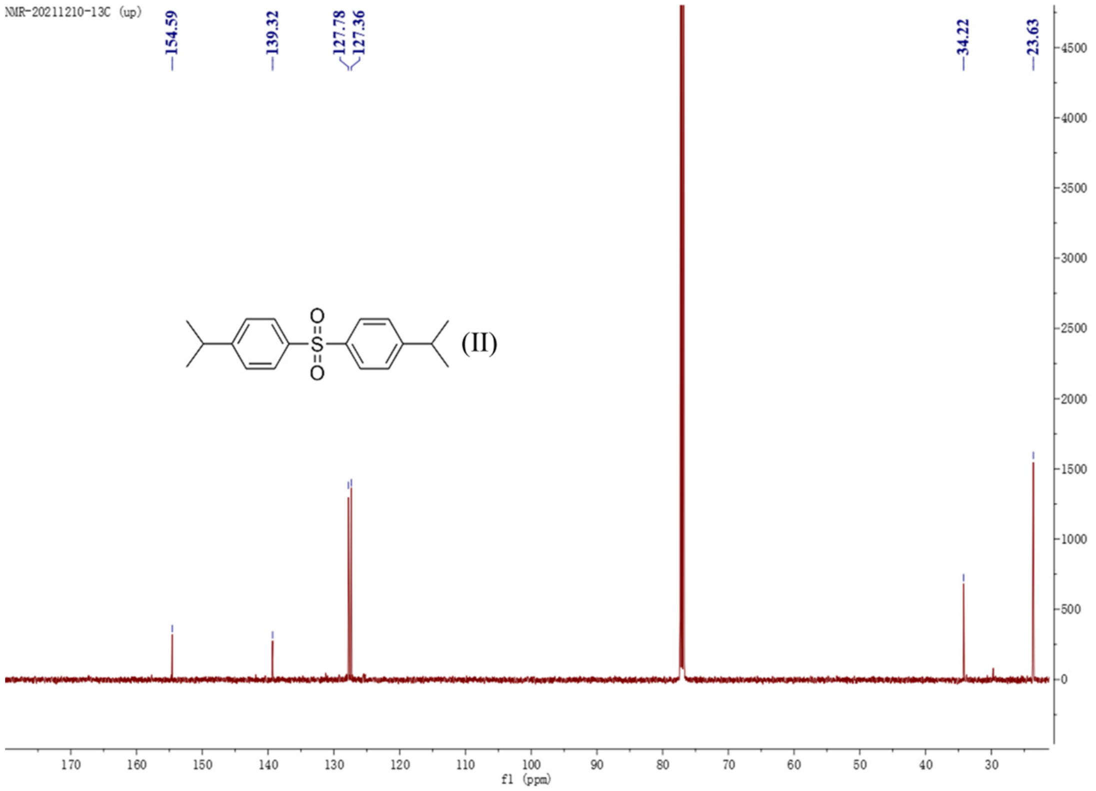

Figure S6. ^{13}C NMR spectrum of the adsorbed product II

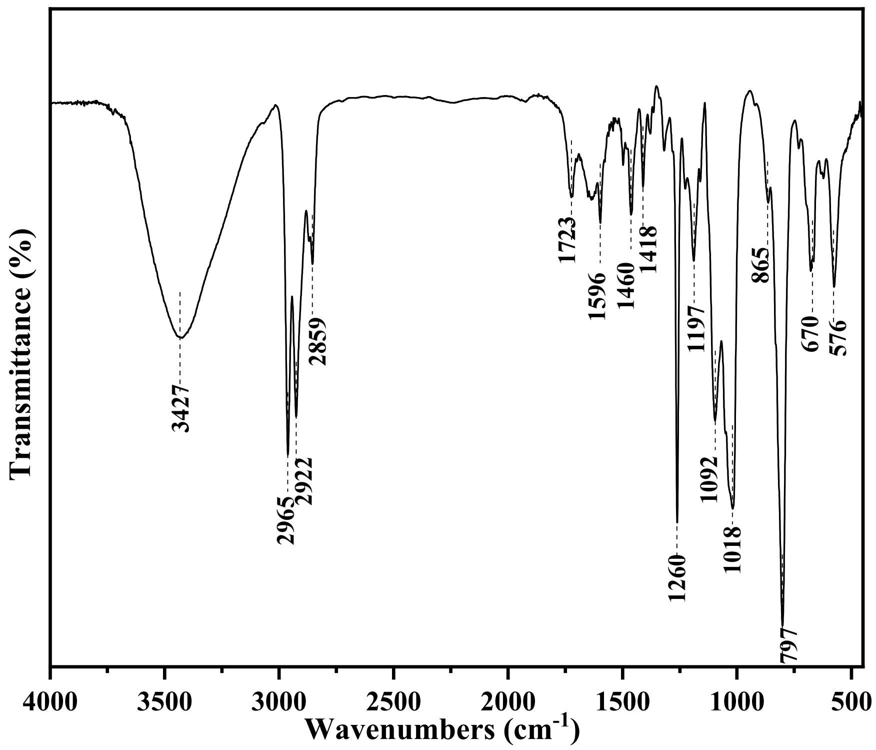

Figure S7. FTIR spectrum of the adsorbed product from the resulting mixture

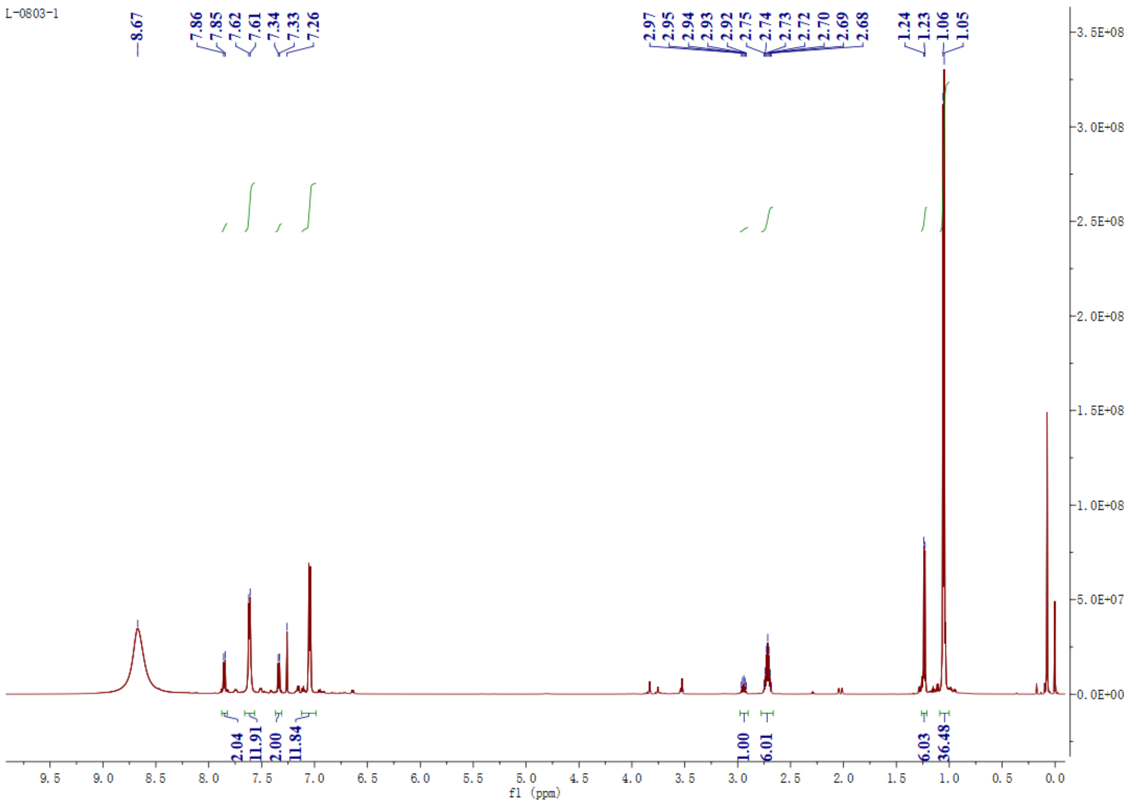

Figure S8. ¹H NMR spectrum of the adsorbed product from the resulting mixture

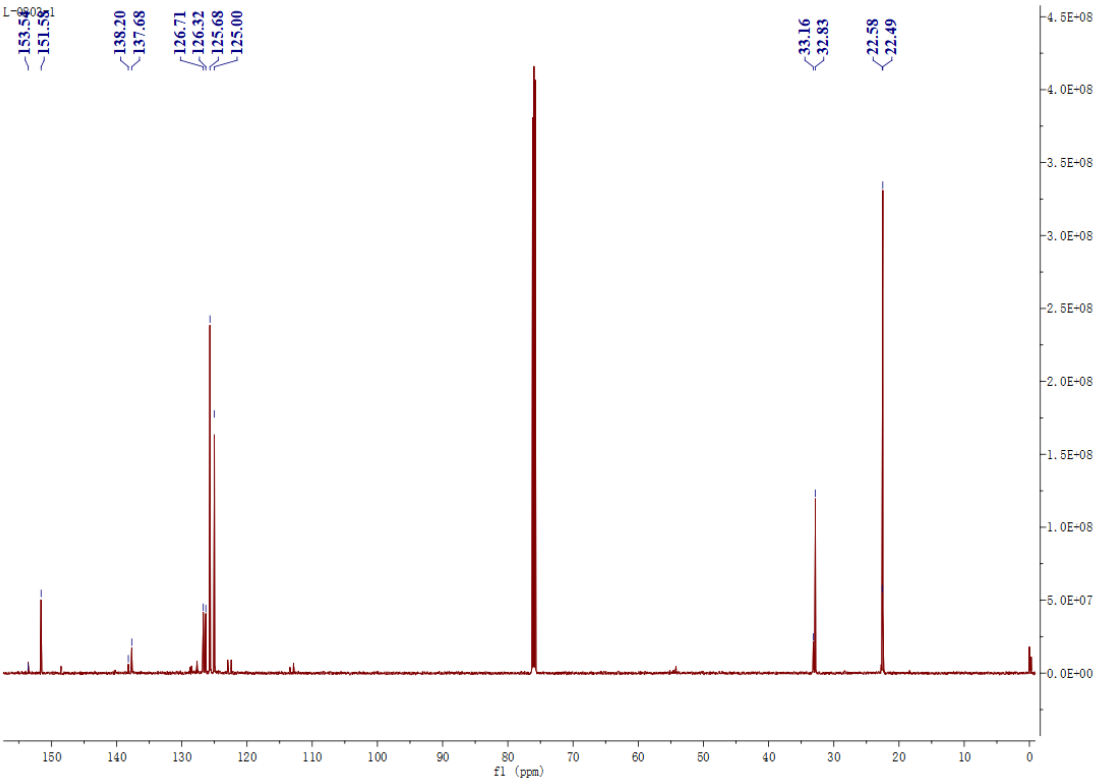

Figure S9. ^{13}C NMR spectrum of the adsorbed product from the resulting mixture

Instruction:

Since there is one cumene residue structure in product I and two cumene residue structures in product II. Therefore, the following formula should be used to calculate the ratio of product I and product II.

$$\frac{N_I}{N_{II}} = \frac{H_I}{\frac{H_{II}}{2}} \quad \text{.........................} \quad \text{(S-1)}$$

Where, N represents the amount of substance (mol) of products I and II, and H is the integral value of hydrogen atom in ^{1}H NMR spectra of the mixture (**Figure S8**).

Table S1. Experimental and fitting results on adsorption performance

Parameters and metrics	Exp. No.						
	1	2	3	4	5	6	7
Experimental:							
t_B (min)	6.34	24.68	56.69	26.04	16.67	69.81	10.68
Q_B (mg g^{-1})	114.44	222.41	259.29	135.11	228.51	316.73	145.17
Dose-response model:							
q_0 (mg g^{-1})	0.324	0.361	0.374	0.264	0.494	0.432	0.299
a	3.569	5.902	8.792	4.695	5.048	9.843	4.033
R^2	0.996	0.999	0.998	0.995	0.999	0.999	0.999
$t_{B,\,th}$ (min)	6.73	24.87	58.06	26.56	16.25	69.60	10.42
$Q_{B,\,th}$ (mg g^{-1})	118.31	223.13	268.55	140.80	231.43	324.50	151.59
